# Immunotherapy and Stereotactic Body Radiation Treatment—An Overview of the Current Landscape of the Strategic Combination of Two Treatment Modalities to Achieve Better Therapeutic Outcomes

**DOI:** 10.3390/cancers18111682

**Published:** 2026-05-22

**Authors:** Aswin Abraham, Anjali Menon, Kurian Joseph, Wilson Roa, Beena Kunheri

**Affiliations:** 1 Department of Radiation Oncology, Cross Cancer Institute, Edmonton, AB T6G 1G1, Canada; anjali.menon@cancercarealberta.ca (A.M.); kurian.joseph@cancercarealberta.ca (K.J.); wilson.roa@cancercarealberta.ca (W.R.); 2 Department of Clinical Oncology, Maidstone and Turnbridge Wells NHS Trust, Kent ME16 9QQ, UK; beenakunheri@yahoo.co.in

**Keywords:** immunotherapy, SBRT, combined treatment

## Abstract

Radiation plays an important role in the treatment of many solid tumors. While conventional fractionation schedules are commonly used, technological advances have allowed safer and more effective dose escalation in the treatment of both primary as well as metastatic lesions. There is growing evidence about the intricate role played by the immune system in modulating the effect of cancer treatment, and several pre-clinical and clinical studies have explored the use of immunotherapy in the treatment of various cancers, both in the metastatic and upfront treatment setting. This approach has shown significant success in the management of melanomas and lung cancers and has prompted exploration of the same strategy in many other cancer subtypes. Radiation has been proposed to synergize the effect of immunotherapy through various mechanisms and is currently being evaluated in various ongoing clinical trials. Several mature clinical studies have also shown significant benefit with the combination and have led to its uptake in routine clinical practice. Furthermore, the use of hypo-fractionated high-dose radiation or Stereotactic Body Radiation Treatment (SBRT) regimens have shown benefits over conventional radiation in this context and are being actively explored as a treatment strategy.

## Simple Summary

The changes in the field of cancer treatment in the last few years have been driven to a large extent by the rapid uptake of immunotherapy for the treatment of various cancers and by the technological revolution in the field of radiation treatment with advanced treatments like high-dose focused radiation being routinely used in the treatment of both primary and metastatic cancers. Although they have been known to be excellent modalities of treatment by themselves, the combination of the two treatments has been hypothesized to work in an additive manner to bring about better treatment outcomes. Several trials are exploring the combination of immunotherapies and SBRT and are likely to be game-changers for the treatment of various malignancies. This review compiles the available data regarding the benefit of combining these treatment modalities in various cancer sub-types and explores some of the ongoing research in this area.

## 1. Introduction

The increasing use of immunotherapies for the management of various cancers has opened the possibility of improved outcomes in the management of several cancers with survival in many patients far exceeding what was seen in the era of non-targeted systemic therapies. Despite the improved outcomes, there is considerable interest in examining approaches to get better treatment responses. Radiation therapy, especially high-dose ablative radiation, has been known to produce an immunostimulatory environment and could potentiate the effect of immunotherapy. The salient information with relevance to oncological immunotherapy, radiation immune effects, and scientific rationales for combining radiotherapy and immunotherapy is discussed in this narrative review.

## 2. Methods

This narrative review was conducted to collate clinically relevant evidence on the combined use of immunotherapy and stereotactic body radiation in the treatment of various solid tumors. A comprehensive search was conducted across PubMed, Google Scholar and ClinicalTrials.org to identify relevant studies. Searches were executed using keywords and subject headings between January and March 2026, including immunotherapy, SBRT/stereotactic body radiation treatment and specific cancer types. Inclusion of reported data was limited to peer-reviewed articles published in English and relevant to the topic, and ongoing trials were included based on relevance and study robustness. Study titles and abstracts of the published data were screened for relevance and full-text articles were evaluated to ensure alignment with this review. Due to the narrative design, no article count or formal bias assessment was conducted.

## 3. Cancer Immunology and Immunotherapy

The immune system plays a vital role in keeping malignant processes in check and is partly regulated by an elaborate, but well-balanced, interaction between Antigen Presenting Cells (APCs) and T-cells, among many other mechanisms. The immune response is regulated by both the presence and the balance of stimulatory and inhibitory signals and their receptors on the effector cells. Stimulatory signals propel a response that will inhibit the neoplastic process from progressing, while the absence of such signals or the presence of inhibitory signaling results in the attenuation of an immune response. A detailed summary of the immune response process is beyond the scope of this chapter, but the salient information with relevance to this topic is discussed.

The physiologic response that recognizes tumor cells and keeps them in check is mediated by several types of immune cells [1] that include the cytotoxic CD8+ and CD4+ T-cells and the helper T-cells that recognize non-self-antigens via interactions with T-cell receptors (TCRs) and Major Histocompatibility Complex (MHC) proteins. APCs play a very important role in this process and mediate the immune response through processing and presenting antigens to the T-cells. Subsets of T-cells like the T-regulatory cells (T-regs) and Myeloid Derived Suppressor Cells (MDSCs) help maintain homeostasis by suppressing the immune response. Other cellular components of this complex mechanism of recognition and disposal of cells undergoing neoplastic processes include the Natural Killer (NK) cells that target cells with low MHC class 1 expression, even in the absence of antigen presentation, as well as the macrophages that are involved in cell clearance and secretion of interferons and cytokines.

The control and regulation of the immune response are essential to avoid any catastrophic activation under normal circumstances, although this can be hijacked by malignant processes to evade the anti-tumor effect of the effector cells. Two essential pathways that play a role in the ‘yin and yang’ of cancer immunology with clinical relevance include the Cytotoxic T Lymphocyte Antigen 4 (CTLA-4) and the Pro-grammed Cell Death Protein 1 (PD-1) pathways. CTLA-4 is an inhibitory regulator of T-cell response and impairs immune activation [2]. It is a homolog of the CD-28 receptor present on T-cells and binds to the same ligands on the APCs, although with higher affinity. CTLA-4 exerts its effects by out-competing CD-28 for binding to CD80 and CD86 on the APCs, thereby attenuating the interaction [3,4,5]. Expression of CTLA-4 is driven by naïve T-cell activation as well as by cytokines like In-terleukin-12 (IL-12) and Interferon gamma (IFN gamma). PD-1 is another important regulator of the immune system and is a transmembrane receptor found on T-Cells, NK cells and B cells. Ligands of PD-1 include PD-L1 and PD-L2, and engagement with the ligands by PD-1 can cause a downregulation of the immune response. These ligands are expressed on many cell types, including tumor cells, and are induced by pro-inflammatory cytokines [6,7] and often upregulated in tumors [8].

While the above pathways have been explored in detail both in the pre-clinical and clinical domains, emerging data on other checkpoints in immune modulation in cancers is gaining prominence. Several T-cell inhibitory receptors, including LAG-3, TIM-3 and TGIT, have gained prominence in this area [9,10]. Myeloid and B-cell regulators like VISTA and BTLA act as negative regulators of T-cell activation. Phagocytosis and metabolic checkpoints like CD47, CD73, CD 38, NKG2A, B7-H6 and HHLA2 are other emerging checkpoint targets that can activate NK and T-cell cytotoxicity against various cancers [11].

During the process of malignant transformation, cells become immunologically distinct from normal cells due to the presence of aberrant antigens and are recognized and eliminated by the immune cells. Cancer cells with a high load of tumor mutational burden are likely to express these aberrant antigens or “neo-peptides” that lead to immune activation [12,13]. This process is enabled by the innate and adaptive immune systems, and when successful, leads to elimination of the cancer. Sometimes, the cancer cells survive this elimination process but are prevented from progressing by the immune system and remain potentially dormant, in a state of equilibrium. Eventually, the cells will escape the control of the immune system and experience progression due to antigen loss, insensitivity to the immune effector mechanisms and potential induction of an immunosuppressive tumor microenvironment. The three components of elimination, equilibrium and escape together form the regulatory process called immunoediting in cancer immunology [14].

An immunosuppressive microenvironment conducive to tumor progression is created by the tumor cells via cytokine secretion or recruitment of regulatory immune cells such as T-regs or MDSCs, which in turn go on to express negative regulators like CTLA-4, PD-1 and PD-L1 to inhibit T-cell activity. In the pre-immunotherapy era, expression of the inhibitory regulators was associated with poor outcomes, including poor overall survival. This has dramatically changed with the introduction of immunotherapies targeting the above-mentioned regulatory pathways or checkpoints [15,16,17]. Immunotherapies using anti-PD-L1 and anti-CTLA-4 checkpoint inhibitor agents have shown dramatic benefits and have become the standard of care for many cancer types. Clinical trials exploring drugs targeting other aspects of the immune milieu like TGF-β are being explored in many solid tumor sites [18].

While a detailed look into the currently available immunotherapeutic agents will be difficult in the context of this chapter, a number of these agents have been approved for use in routine clinical practice and many more are in the clinical trial phase. All of these promise significantly improved outcomes in a number of cancer types.

## 4. Radiation and Its Immune Effects

The principal mechanism of action of radiation is its effect on the cellular DNA, with treatment eventually resulting in cell death via various processes like apoptosis, mitotic catastrophe, necrosis and autophagy, among many others. Radiation treatment can also promote various immune processes, with the potential to lead to an immune-mediated anti-cancer effect. These effects may be localized and restricted to the micro-environment in proximity to the treated area or may lead to an enhancement in immune recognition and improved immune-mediated tumor control at distant sites known as an “abscopal” effect [19]. Several mechanisms have been proposed to be behind the immune response and may in turn be modulated using immunotherapies [20,21,22,23,24,25,26]. Radiation leads to increased cell kill following DNA damage and by extension promotes increased antigen spillage and antigen presentation. Radiation can upregulate immunogenic cell surface markers including major histocompatibility class I (MHC class I) molecules, which in turn help T cells recognize foreign peptides. By way of secretion of stimulatory chemokines and cytokines, there is active recruitment of T-cells to the tumor sites and there is also increased lymphocyte infiltration into the tumor stroma. An upregulation of PD-L1 is also noted on the cancer cells, as well as activation of the apoptotic Fas/FasL and other pathways, that modulate the activity of T-cells and elimination of the tumor cells through T-cell and other immune responses. Activation of Natural Killer cells (NK cells) via NKG2D expression is another non-T-cell-mediated immune response promoted by radiation. All of these work synergistically to promote immune recognition and tumor cell kill at the local site and are also thought to elicit the abscopal effect.

## 5. Stereotactic Radiation and Its Immune Effects

Despite its significant anti-tumor effects, radiation treatment has been found to be a double-edged sword, with prolonged treatment associated with reducing returns in terms of immunological response [27,28,29]. Immune effector cells are very sensitive to radiation [30] and repetitive killing of these cells during repeated radiation treatments, as would be the case with conventional radiation, is associated with reduced immune response and poor overall outcomes [27,31,32,33]. In this context, despite the higher dose per fraction, stereotactic body radiation treatment (SBRT) may have less of an impact on the immune response due to fewer treatment fractions and, consequently, less exposure of the blood pool to radiation [34,35]. Furthermore, SBRT is also thought to have other advantages due to its impact on the micro-environment and immune activation. SBRT increases T-cell infiltration into tumors due to the greater degree of stromal and vascular damage that promotes extravasation into the tumors [36]. An increase in tumor kill may also be associated with greater antigen spillage, antigen recognition and antigen presentation to effector cells, all of which, in turn, may be associated with improved outcomes [37]. Another interesting finding with the use of SBRT is that of the tumor debulking effect and the improvement in immune response. A rationale proposed for this effect is that tumors constantly produce inhibitory signals that lead to T-cell exhaustion [38]. The reduction in tumor burden is in turn believed to take away the inhibitory signaling and restore T-cell function, thereby improving the anti-tumor immune response.

In spite of the theoretical benefit of SBRT for immune activation, there has been conflicting clinical data as well in this area. The abscopal effect of SBRT is controversial as out-of-field systemic responses remain unpredictable in many clinical scenarios. Also reported in the literature is the paradoxical effect that high-dose SBRT can have on the immune-activating property of SBRT. Pre-clinical and early clinical data appear to show an upregulation of the TREX1 enzyme in cancer cells, which in turn causes an upregulation of the cGAS/STING pathway leading to degradation of free-floating DNA that would have played a role in immune activation [39]. Another area that is also relevant to the arena of SBRT is the immunological differences between a single- versus multi-fraction SBRT. Single large doses can produce immunostimulatory effects like multi-fraction SBRT, as seen in phase II data in the metastatic setting (SAFRON II trial-NCT01965223) [40], and actively increase stimulation of CD 8+ cells but can potentially reduce the proportion of “exhausted” PD-1 positive T-cells. A single fraction of high-dose radiation may also lead to increased vascular endothelial cell damage with arguments both for and against this effect, where endothelial cell damage may come with increased tumor disruption, while on the other hand a more appropriate benefit would be from lower radiation doses producing improved vascular normalization leading to better oxygenation and tumor response to radiation [41,42]. A high-dose single-fraction approach also comes with the TREX1 “paradox” noted above. On the other hand, multi-fraction SBRT can potentially perform better in inducing immune-mediated abscopal effect [43] and may also be better tolerated in actual clinical practice [44]. An in-depth analysis of the differences between the single and multi-fraction SBRT will be beyond the scope of this review.

## 6. Rationale for Combining Stereotactic Radiation and Immunotherapy

**Figure 1:**
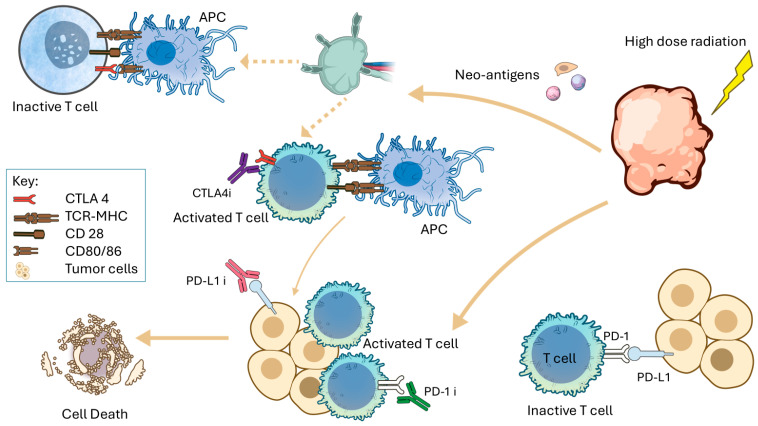
The synergistic effects of the combination of high-dose radiation and checkpoint inhibitors. PD 1—Programmed Death 1, PD-L1—Programmed Death Ligand 1, APC—Antigen Presenting Cell, CTLA-4—Cytotoxic T Lymphocyte Associated protein 4, CTLA4i—CTLA4 inhibitor, PDi—PD inhibitor, PD-L1i—PD-L1 inhibitor.

While radiation treatment alone may indeed lead to a systemic immunological response, this is presumed to be rare and the data available is generally restricted to anecdotal evidence and small early-phase clinical trials [45,46]. On the other hand, the advent of immunotherapies has indeed heralded a new era of improved survival in several cancer types, but the evolutionary pressure from the tumor cell killing by the immune system gradually confers on the cancer cells the ability to evade the response to these treatments. Thus, strategies to overcome these immune resistance mechanisms are needed for continued treatment effectiveness. One such strategy being increasingly explored is that of combining immunotherapies with radiation. This has been reported to be very promising in terms of the improved response to treatment, and there is growing evidence supporting this approach (Figure 1).

The synergistic effects of SBRT and immunotherapies are multi-faceted. The increase in tumor cell kill following radiation and the subsequent antigen spillage result in increased antigen presentation and recognition by the immune effector cells [37]. This process works in complement with PD-L1 and CTLA-4 inhibitors—by far the most used immunotherapy or checkpoint inhibitory agents—and has shown excellent response in different solid tumors [47,48]. Radiation upregulates MHC class I expression as well as Calreticulin and HMGB1 expression [49], and this process has been shown to improve tumor response in pre-clinical studies with the combination of radiation and immunotherapy [25]. Along with the above-mentioned cell surface proteins, cell surface PD-L1 expression is noted to increase because of radiation, making the cells susceptible to checkpoint inhibitors [50]. Tumor debulking with SBRT may also play an important role in improving the therapeutic response of immunotherapy with the combination of SBRT. The reduction in tumor burden is thought to remove the inhibitory mechanisms that may in turn improve the therapeutic response of immunotherapy [38]. The abscopal effect is yet another mechanism thought to play a role in enhanced disease control. Pre-clinical studies have shown an abscopal effect with the strategy of combining radiation with immuno-therapeutics [43], and this is believed to be dependent on the tumor-related antigens released from the treated site. A recently reported phase I study of SBRT followed by Pembrolizumab in metastatic solid tumor patients appears to validate the idea of the abscopal effect, with non-irradiated tumors showing a response with a measurable reduction in size [51]. The concept of improved antigen spillage and presentation with multi-site treatment has also been explored, and currently, along with mono-site SBRT, there is also evidence supporting multi-site and multi-fraction radiation treatment for improved radiation and immunotherapy effects [52].

Despite all the hypothesized effects with the combination strategy, not all clinical trial data support this approach, with conflicting reports about the true clinical benefit [53]. In actual clinical practice, while some studies appear to show excellent results with the combination of SBRT and immunotherapy, other phase 3 trials have failed to show a statistically significant survival benefit. Certain factors like the optimal timing or sequencing of SBRT and immunotherapy are also hard to reconcile with the current level of evidence [54]. In short, while SBRT may create a favorable, “inflamed” microenvironment that may be exploited by combination with immunotherapies compared to conventional radiation, this strategy remains highly debated and quite likely tumor-dependent.

Although there are nuances to the relevance of this approach, the combination strategy of radiation and immunotherapy has been applied in clinical practice (Table 1 and Table 2). It involves major cancer sites including lung cancer, oligo-metastasis, melanoma, and genitourinary, gastrointestinal, and breast cancers.

## 7. Hyperthermia—A Novel Therapeutic Approach to Improve Effectiveness of the Combination of SBRT and Immunotherapy

Although immunotherapeutic agents are quite effective in relieving the blockage of immune response to various cancer types, they are not very effective in “cold” tumors that are poorly infiltrated by immune cells [76]. Hyperthermia and immunotherapy can work synergistically through modulation of the tumor microenvironment, enhancement of immune cell infiltration and induction of cell death [77,78]. Similarly, the combination of hyperthermia and radiation results in synergistic effects including the release of damage associated proteins that in turn activate T-cells that promote leucocyte adhesion and infiltration, enhancing blood flow and creating a favorable microenvironment favoring better immune cell infiltration [79]. The strategy to combine the three modalities—though complex, may make a significant improvement in the outcomes of otherwise hard-to-treat cancers and are being actively explored.

## 8. Lung Cancer

Arguably, some of the greatest impact that the combination strategy of radiation and immunotherapy has produced in clinical practice has been in the treatment of lung cancers. Immunotherapy has become intricately intertwined with the management of non-small cell lung cancer (NSCLC) [80]. Antibody therapies targeting the CTLA-4, PD-1 and the PD-L1 pathways have been approved in various treatment settings in lung cancer patients and are currently routinely used in clinical practice. But immunotherapy by itself has had mixed success in advanced lung cancers. In comparison with chemotherapeutic agents, the use of the PD-1 inhibitor Nivolumab failed to show a difference in survival when used in the locally advanced and metastatic setting in patients with ≥5% PD-1 expression [81] and similar results were noted with the use of the CTLA-4 inhibitor Ipilimumab, when used in combination with paclitaxel and carboplatin chemotherapy in advanced NSCLC [82]. In contrast, the use of Pembrolizumab, a PD-1 inhibitor, in the phase 3 KEYNOTE-24 study was associated with a significant improvement in the overall survival (OS) and progression-free survival (PFS) in patients with PD-L1 expression ≥50%, when compared to standard chemotherapy [83,84,85]. Another landmark study that changed the lung cancer treatment paradigm was the PACIFIC trial that evaluated the adjuvant use of the PD-L1 inhibitor Durvalumab after definitive chemoradiation. A significant progression-free survival (PFS) was noted with this approach [86] and is currently the standard of care for locally advanced lung cancers. The use of Atezolizumab, another PD-L1 inhibitor, was also associated with improved survival in the setting of previously treated metastatic lung cancer [87]. Previously untreated metastatic non-squamous NSCLC treated with Pembrolizumab in addition to standard chemotherapy with Pemetrexed and platinum was evaluated in the phase 3 KEYNOTE-189 study and showed significantly improved 1 year survival of 69.2% in the immunotherapy arm versus 49.4% in the comparator arm [88]. While these are impressive outcomes, the overall success of these immunotherapeutic agents is plagued by the variable and sometimes short-lasting response, and it is in this context that the addition of radiation may play a significant role. Some of the first clinical evidence in this context was noted in the KEYNOTE 1 study, where patients who had received radiation prior to Pembrolizumab were noted to have a significantly better PFS and OS [89]. This in fact paved the way for some of the subsequent studies in this area.

Along with NSCLC, the management of small cell lung cancer (SCLC) has also significantly evolved over the last few years. Chemoimmunotherapy has become the standard of care for extensive stage SCLC (ES-SCLC) based on the phase 3 KEYNOTE 604 and Impower 133 studies that showed an improvement in overall survival and progression-free survival (12 month PFS was 13.6% with immunotherapy vs. 3.1% with chemo in the KEYNOTE 604; 13.9 month PFS was 14.9% with immunotherapy and 6.4% with chemo in the Impower 133) [90,91]. SBRT is also being more commonly used in the domain of SCLC, especially in the limited stage SCLC group of patients. Based on the postulation that the synergy between SBRT and immunotherapy can be utilized for treatment of SCLC, the phase 2 NCT02701400 study evaluated the utility of the use of the PD 1 inhibitor Durvalumab and the CTLA-4 inhibitor Tremelimumab with SBRT in relapsed SCLC patients. Although no survival advantage was noted, there was a trend towards efficacy [92]. This has supported the setting up of several other studies that look at the combination of SBRT with immunotherapy in the management of SCLC.

### 8.1. Extracranial Lung Cancer Oligo-Metastases

The combination of SBRT with immunotherapy for NSCLC has been explored in the oligometastatic setting in a phase II single-arm study where SBRT, conventional radiation, local ablative therapy and surgery followed by Pembrolizumab were evaluated (NCT02316002). There was a significant improvement in the PFS (19.1 months compared to 6.6 months) and OS (41.6 months) in these patients when compared to historical controls [55]. This data showing significant improvement compared to previous evidence with immunotherapy alone led to further studies with this treatment approach. The phase 2 PEMBRO-RT trial evaluated patients with advanced NSCLC who were randomized to receive Pembrolizumab with or without SBRT to a single metastatic site [56]. A separate non-irradiated tumor was evaluated for objective response rate (ORR). The study noted a significant improvement in ORR (36% vs. 18% for the investigational and control arms, respectively) for the patients who received SBRT. A non-significant trend towards improvement in PFS and OS was also noted that was attributed to the group of patients with low PD-L1 expression. This study also highlighted the need to identify the group of patients who are most likely to benefit from the combination treatment. Another phase I-II study evaluating concurrent versus sequential Ipilimumab and SBRT for metastatic NSCLC was also recently reported and noted better outcomes in patients receiving sequential Ipilimumab after SBRT, strengthening the argument about the importance of treatment sequencing [57,58]. The ongoing NRG-LU002 study (NCT03137771) exploring local ablative therapy and maintenance therapy (including immunotherapy) and the LONESTAR study (NCT03391869) evaluating induction immunotherapy followed by local consolidative therapy will potentially answer some of the questions raised regarding the utility of the combination therapy for extracranial lung cancer oligo-metastases. While not specific for extracranial metastases, the recently opened BR.38 (NCT06686771) trial evaluates the use of SBRT and immunotherapy in patients with oligo-progressive metastatic lung cancer and likely will give more information regarding the combination approach in the target population who otherwise has limited management options.

In the ES-SCLC arena, there is limited prospective data that has been reported on the combination of ablative therapy and immunotherapy. While studies like the NRG LU007 (NCT04402788) evaluate the role of consolidative radiation after chemo and immunotherapy, the DARES study (NCT05068232) will assess the role of SBRT along with systemic chemoimmunotherapy [93].

### 8.2. Lung Cancer Brain Metastases

The occurrence of brain metastases is an unfortunate complication of advanced NSCLC and is associated with poor outcomes [94]. Surgical resection and local treatments like stereotactic radiosurgery (SRS) and SBRT, as well as whole-brain radiation, remain the mainstay of treatment for these patients with brain metastases. The use of local treatments like SRS and SBRT is thought to produce local tumor cell kill, as well as increase the permeability of the blood–brain barrier, which in turn leads to better drug penetration [95]. Pre-clinical studies have also shown a radiation-induced, interferon-γ-mediated, upregulation of vascular cell adhesion molecule-1 (VCAM-1) on vasculature and MHC-1 markers on the cancer cells [96], all of which are thought to increase the potential for increased immune activity in the metastatic lesion. These factors thus support the potential use of radiation and immunotherapy in the management of brain metastases from lung cancers.

Unfortunately, there are limited prospective studies that have explored this clinical setting. Even with the available retrospective data, the definition of concomitant treatment differed between studies, making it difficult to come to valid conclusions. A retrospective analysis evaluating patients with brain metastases from NSCLC showed that the combination of checkpoint inhibitors with SRS/SBRT was associated with improved local control and OS, as well as a decrease in distant brain failure and risk of neurologic death [97]. A matched cohort study looking at a similar group of patients also reported that the concurrent use of immunotherapy and SRS was well tolerated and provided rapid disease regression in the brain [98]. A recently reported meta-analysis that combined the data from the few available studies exploring the question of brain metastases in NSCLC patients further explored the question of the benefit of combining immunotherapy with radiation and reported improved local disease control and OS outcomes without a significant increase in toxicity [99]. Thus, while being considered an evolving field, the existing data appear to support the combination treatment approach for brain metastases from lung cancers.

A few prospective studies are in fact exploring this question and are likely to give us good insight in the near future. The phase 2 STICk-IM-NSCLC study (NCT04650490) is currently evaluating the benefit of stereotactic radiosurgery and immunotherapy for untreated brain metastases, and the STRAITLUC (NCT04787185) study is evaluating fractionated stereotactic radiation along with immunotherapy in a similar setting. The MIGRAINE study (NCT04427228), on the other hand, is a randomized trial that will evaluate single versus multi-fraction SRS along with immunotherapy for brain metastases, although the inclusion criteria will include non-lung primaries as well.

### 8.3. Early-Stage/Non-Metastatic Lung Cancer

SBRT has been accepted as a standard of care treatment for early-stage lung cancers, especially in the medically inoperable group of patients. While acknowledging the risk of distant failure, it has been reported to be associated with significant local disease control in early-stage lung cancers [100]. Early-stage lung cancer patients generally do not receive systemic treatment after local treatment, leaving them at a low, but potentially increased risk of distant failure [101]. The data from the PACIFIC and KEYNOTE studies as well as the PEMBRO-RT study that combined the use of radiation and immunotherapy in the setting of locally advanced and advanced lung cancers revealed significant improvements in the response rates and survival outcomes and raised the question of whether these same benefits may extend into the group of patients who have early-stage lung cancers.

Given the widespread use of SBRT for the treatment of early-stage lung cancers and the potential for a more immunogenic response to SBRT compared to conventional radiation, the belief is that the addition of immunotherapies to the treatment regimen may be associated with improved survival outcomes and a change in the patterns of failure in early-stage lung cancers. The PACIFIC-4 (NCT03833154) and KEYNOTE-867 (NCT03924869) are phase 3 trials exploring the use of SBRT followed by Durvalumab and SBRT with concurrent and adjuvant Pembrolizumab respectively, in the treatment of early-stage lung cancers. Although the PACIFIC-4 is ongoing, the KEYNOTE-867 trial was discontinued due to a lack of recurrence-free survival benefit at the time of an interim review [59]. The I-SABR (NCT03110978) and the ASTEROID (NCT03446547) are phase II studies exploring SBRT along with Nivolumab and Durvalumab, respectively, in a similar setting. The I-SABR trial reported that compared to stereotactic radiation alone, immunotherapy along with SBRT improved event-free survival at four years [60]. A first report from the ASTEROID trial showed that the combination of SBRT and Durvalumab was well-tolerated [61]. The primary end point of time to progression is yet to be reported. Interestingly, the primary endpoints are examined differently for each study but are expected to give significant insight into the benefit of the combined treatment. Only time can reveal if this is a truly relevant and cost-effective approach for the management of early-stage lung cancers.

Overall, the use of the combination strategy of SBRT and immunotherapy appears to be beneficial in the management of locally advanced and metastatic lung cancer and less so in the early-stage lung cancers, although further studies that are ongoing and maturing will help clarify the true benefit of this approach.

## 9. Melanoma

Melanomas are immunogenic tumors, with most being diagnosed at an early stage. In the patients who present with metastatic disease or develop metastases, various systemic agents have been tried with varying success. High-dose interleukin-2 was possibly one of the first immunotherapeutic agents that was noted to have significant benefits in these patients, although the toxicity associated with it severely limited its use. The development of checkpoint inhibitors and targeted agents like BRAF and MEK inhibitors has significantly improved survival outcomes in the recent past and are routinely used in the metastatic setting.

Locally advanced melanomas are usually treated with checkpoint inhibitors with significant treatment effect, whereas unresectable localized lesions may be treated with radiation. Interestingly, the studies evaluating the combination of radiation with the checkpoint inhibitors for the treatment of locally advanced melanomas are limited. Retrospective studies have shown an improvement in survival with the combination of Ipilimumab and radiation [102] and have subsequently led to early-phase clinical trials exploring the question of efficacy prospectively. The phase II NCT02821182 study evaluated Nivolumab and SBRT in melanomas and included 35% of patients with locally advanced disease. The study noted a response rate of 45% which was similar to previous reports of the use of checkpoint inhibitors alone, but circulating tumor DNA analyses suggested that certain patients responded to treatment only after receiving SBRT [62]. The NCT01557114 was a phase 1 study evaluating Ipilimumab and radiation in locally advanced melanomas. The study showed improvement in antitumor activity represented by the improved response rates [63].

Brain metastases occur in about 10–40% of the patients with advanced melanoma. While surgery and radiation remain the treatments of choice, the introduction of checkpoint inhibitors has opened the discussion about a synergistic effect between these medications and radiation. Several retrospective and early-phase studies have reported on the outcomes with the combination of checkpoint inhibitors and radiation [103]. The local control in the studies ranged between 45 and 92.3% for SRS alone, while the combination treatment of SRS and checkpoint inhibitors was associated with local control rates ranging from 16.5% to 100%. The results, unfortunately, have not been uniform, with some studies showing significant local control benefits with the combination strategy, while others showed no such advantage. The ABC-X study (NCT03340129) is a phase II study currently randomizing patients with melanoma brain metastases to either Ipilimumab and Nivolumab alone or in combination with SBRT to the brain lesions. This and other upcoming studies should shed light on the benefit of the combination treatment in melanoma brain metastases.

Soft tissue/visceral and bone metastases are also common in advanced melanomas, and the combination of radiation and checkpoint inhibitors has been examined in this context as well, with variable outcomes [104]. Improved overall survival was noted in some studies [105], which was attributed to an extent to the abscopal effect of the combination therapy [106]. Liver metastases are particularly susceptible to the combination treatment and are believed to be due to the significantly higher immunogenic effect noted with treatment at this site. The recently completed HAMMER study (NCT05169957) specifically looked at hepatic melanoma metastases to evaluate the feasibility of immunotherapy with Ipilimumab and Nivolumab and the combination with SBRT, with a view to identifying the enhancement of immune response following treatment of liver metastases.

Despite the limitations of many of the retrospective studies and lack of current data on prospective studies, the overall picture is that of good local control and appreciable distant response rates with the combination of immunotherapy and SBRT in this tumor subgroup.

## 10. Genito-Urinary Malignancies

### 10.1. Renal Cell Carcinoma

The management of renal cell carcinoma (RCC) has evolved significantly in the last few years, with the increased understanding of the disease itself and the biological pathways that modulate disease progression. The introduction of immunotherapy in the advanced renal cell carcinoma setting has significantly changed the landscape of treatments available for this disease, and the immunotherapy agents are currently used either as monotherapy or along with other treatments for the management of advanced RCC. The results from combination therapies have been controversial, with studies having differing inclusion criteria for patient groups and differences in measuring treatment response between studies. In spite of this, the current checkpoint inhibitors do appear to benefit advanced RCC patients, with the most benefit likely for the clear cell and the intermediate/poor-risk group of RCCs.

RCC has traditionally been considered to be resistant to treatment with radiation. But increasing doses and high accuracy, as is seen with SBRT, are being regularly used for the treatment of metastatic lesions. SBRT has been explored with significant success in the treatment of brain and extracranial RCC metastases, and there is even evidence from early-phase trials regarding the utility of SBRT for the management of the primary renal lesion. Some key immune responses discussed above are triggered by high-dose radiation of RCC tumors and can act synergistically with immunotherapy in the treatment of RCC. This makes the combination of the two an attractive prospect for the treatment of advanced RCC. Unfortunately, prospective evidence supporting this approach in RCC is limited. An early study evaluating the combination of SBRT and Interleukin-2, a pre-checkpoint inhibitor era immunotherapeutic agent, in patients with metastatic RCC noted improved response rates in patients treated with the combination, but did not translate to improved outcomes with longer follow-up [107,108]. A few case reports have suggested improved outcomes with the combination of the newer checkpoint inhibitors and this has prompted the setting up of a number of clinical trials that are exploring the combination of SBRT and immunotherapy in advanced RCCs. Early results from two studies examining checkpoint inhibitors and SBRT appear to show conflicting results. NIVES (NCT03469713) was a study evaluating the efficacy and safety of the combination of Nivolumab and SBRT for pre-treated metastatic RCC. Although early results suggested good disease control rate and objective response rate with no increase in toxicity, a more detailed analysis failed to show a translation of early response to meaningful survival outcomes [64]. RADVAX RCC (NCT03065179), a study evaluating dual immunotherapy with Nivolumab and Ipilimumab, along with SBRT, showed good disease control at the treated site with a reasonable survival outcome [65]. Compared to the former study, this study used a higher radiation dose, which could explain some of the local treatment effects. The RAPPORT trial (NCT02855203), on the other hand, was a single-arm phase I/II trial that evaluated the benefit of total metastatic irradiation with SBRT followed by anti-PDL1 therapy with Pembrolizumab. The trial showed excellent control of disease at known sites with a subset of patients free from relapse even in the long term [66]. The NCT02781506, NCT02855203 and NCT02599779 are some of the other phase II trials evaluating the combination of checkpoint inhibitors and SBRT in the treatment of advanced RCC. The results from the same are awaited, due to the potential for enhanced treatment effect and improved disease outcome with this treatment strategy.

### 10.2. Prostate Cancer and Urinary Bladder Cancer

The combined SBRT and immunotherapy strategy for the treatment of non-RCC genitourinary malignancies is relatively new, and there are very few trials that have reported data in this clinical setting. The two treatment modalities have been independently studied to various extents in prostate and urinary bladder cancers, and currently, a number of clinical trials are exploring the combined treatment with SBRT and immunotherapy in both these tumor types, primarily in the advanced/metastatic setting.

The use of SBRT in the management of oligometastatic disease in prostate cancer has been promising, as was noted in the SABR-COMET (NCT01446744) [109] and the ORIOLE (NCT02680587) [110] studies. The ORIOLE study also noted an enhanced systemic immune response following treatment, with increased T-cell clonotypic expansion when compared to baseline. On the other hand, immunotherapy also appears to show reasonable outcomes in metastatic prostate cancers. Sipuleucel-T, an FDA-approved autologous active cellular immunotherapy agent, prolonged survival among metastatic castration-resistant prostate cancer patients [111]. But similar outcomes with the use of checkpoint inhibitors have been less successful, with several studies showing variable results. Part of this may be attributed to the low tumor mutation burden noted in prostate cancers and consequently, the low T-cell activation and infiltration into the tumors. This would in turn translate to a poor response to immune checkpoint blockade. A few ongoing trials are exploring the use of the checkpoint inhibitors in combination with other strategies to improve disease outcomes, especially in the metastatic setting. In pre-clinical studies, the combination of anti-CTLA-4 antibodies with radiation has reported synergistic effects, and based on this idea, a phase I/II study examined escalating doses of Ipilimumab with or without radiation in metastatic prostate cancer. Among the evaluable patients, about 25% had a good treatment response [112]. The CA184-043 (NCT00861614) study evaluated Ipilimumab against placebo following radiotherapy in 799 metastatic castration-resistant prostate cancer patients. Although the median overall survival was initially noted to be similar [67], longer follow-up showed improved overall survival at all time points in the Ipilimumab arm [68]. More prospective studies will be required before questions about the true utility of the combination therapy with radiation in prostate cancer can be clarified. The POSTCARD GETUG P13 study (NCT03795207) is evaluating SBRT and Durvalumab in the setting of oligometastatic relapse, while the TALON study (NCT04569461) had proposed the use of Pembrolizumab along with SBRT for the treatment of localized disease, but was subsequently withdrawn.

The evidence for the combination of SBRT and immunotherapy is even more limited in the setting of bladder cancers. Early-phase studies like the PLUMMB (NCT02560636) and the NCT03620435 evaluating the safety of the combination of immunotherapy and hypofractionated radiation, reported significant dose-limiting toxicities. Although a few trials are evaluating immunotherapy and radiation for bladder cancers, most of these involve conventional or moderate hypofractionation schedules. The ASTRA trial (NCT07413523) is one of the few prospective studies exploring the use of SBRT in this cancer subtype and will evaluate the benefit of metastasis-directed SBRT along with systemic treatment, including immunotherapy. Future studies examining SBRT and immunotherapy in the setting of bladder cancers will be required to understand the potential utility of this strategy.

## 11. Gastro-Intestinal Malignancies

### 11.1. Liver-Hepatocellular Carcinoma

SBRT is being increasingly used as an ablative modality for the treatment of hepatocellular carcinomas (HCC), especially when curative resection is difficult or medically contraindicated. SBRT plays an important role as a monotherapy or in combination with other local therapies, as well as a bridge to liver transplant in the optimal candidate. Excellent therapeutic outcomes have promoted the role of SBRT in this setting from an ancillary role to a more mainstream role. But the quest for better outcomes persists, and the fact that HCC is an “inflammation-driven” malignancy has encouraged the use of immunotherapy along with SBRT.

Pre-clinical studies have shown encouraging results with the use of checkpoint inhibitors in HCC mouse models, with and without the combination of SBRT [113,114]. The subsequent phase I/II CheckMate-040 (NCT01658878) trial showed durable objective response rates and OS [115]. The CheckMate-459 (NCT02576509), KEYNOTE-224 (NCT02702414) and KEYNOTE-240 (NCT02702401) [116,117,118] studies that followed further verified the utility of checkpoint inhibitors in the treatment of advanced HCC and prompted the accelerated approval of Nivolumab and Pembrolizumab use in clinic. The HIMALAYA (NCT03298451) trial also looked at the use of Tremelimumab and Durvalumab and found that the benefits were non-inferior to the previous generation of Tyrosine Kinase Inhibitors, with a signal towards survival benefit. Unfortunately, despite the positive signals from the studies mentioned, the response rate with the checkpoint inhibitors has not been dramatic and has prompted efforts to find strategies that can increase the therapeutic outcomes.

Despite the evidence supporting the immune activation by radiation and the potential synergistic effect with checkpoint inhibitors, there is very little published data on the combined treatment strategy. Although case series [119] and retrospective analyses as well as some phase I/II trials [69,70,120,121] have reported on the combined treatment approach, there is limited data available in the public domain from large, well-planned prospective randomized studies. But there have been encouraging signals noted in the retrospective and early-phase studies with the combination approach. One report from Hong Kong comparing the use of SBRT alone versus SBRT and immunotherapy showed that patients who received the combination treatment had an improved one- and three-year overall survival [122]. The same group also reported that the combination of locoregional therapy and immunotherapy was associated with a clinical complete response (46%) and with >75% of these patients surviving at three years [123]. Results from the PEMRAD (NCT03316872) trial evaluating the use of SBRT with Pembrolizumab, which were reported recently, showed that the combination of SBRT and pembrolizumab demonstrated a high response rate as second-line therapy for advanced HCC [69]. The START-FIT (NCT03904927) trial evaluated the benefit of sequential transarterial chemoembolization and SBRT followed by immunotherapy as a means of downstaging patients to make them eligible for hepatectomy. The study noted a 42% radiologic complete response in patients receiving the combination treatment [71].

Fortunately, several new trials are currently ongoing and will likely report their preliminary data in the near future. The NCT03482102 trial combines Tremelimumab and Durvalumab with SBRT for advanced HCC or biliary tract cancer. The recently opened NRG GI 012 (NCT07166406) study is also evaluating the benefit of the combination of immunotherapy with SBRT in advanced HCC with the additional factor that these patients will have macrovascular invasion.

Hepatocellular carcinomas thus currently appear to be one area where there is a signal towards improved outcomes with the combination of SBRT and immunotherapy, although a thorough evaluation of the ongoing trials will need to be done to justify making this a routine clinical practice strategy.

### 11.2. Pancreatic Cancer

Pancreatic cancers are known to have poor immunogenic microenvironments, and this has led to poor response to anti-PD-L1 and anti-CTLA-4 monotherapies. Hence, there has been a lot of work exploring strategies to improve the response to the immunotherapeutic agents. In this context, radiation has significant potential and is a strategy being keenly studied. A recent phase II study evaluating SBRT with Pembrolizumab and Trametinib compared to SBRT and gemcitabine showed improved OS in the SBRT + immunotherapy group (14.9 vs. 12.8 months), even though the toxicity was slightly higher in this group [124]. The NCT03767582 was a trial that combined SBRT with Nivolumab, a CCR2/CCR5 dual antagonist and GVAX pancreas vaccine. The phase I component of the study showed that the combination was safe and the phase II results are awaited [125]. The NCT05088889, on the other hand, was a pilot study that evaluated maintenance Ipilimumab and Nivolumab after completion of induction chemotherapy and SBRT in stage 4 pancreatic cancer patients. The study showed excellent disease control rates (90%), although much larger study sets will be required before meaningful benefit can be attributed to the treatment regimen. The INFLUENCE (NCT05116917) trial evaluated immunotherapy combined with SBRT and influenza vaccine for previously treated pancreatic adenocarcinoma patients. Although the combination was noted to be feasible, the interim analysis of the study did not show objective responses and hence did not proceed to complete accrual [126].

In spite of limited data supporting the combination of SBRT with immunotherapy, there have been enough signals noted in previous early-stage clinical studies that have supported the development of further trials in this difficult-to-treat malignancy. The EMPIRE trial (NCT06843551) is currently evaluating the benefit of SBRT followed by dual checkpoint inhibition for metastatic pancreatic cancer, and the PREOPANC-5 (NCT06384560) is evaluating the benefit of the triple therapy of mFOLFIRINOX, Pembrolizumab and SBRT for borderline resectable pancreatic cancer.

### 11.3. Colorectal and Other Gastro-Intestinal Cancers

Check-point inhibitors have demonstrated significant efficacy in select colorectal cancer patients. The KEYNOTE-016, KEYNOTE-164 and KEYNOTE-177 studies showed significant benefit with checkpoint inhibitors when treating mismatch repair (MMR)-deficient colorectal tumors. Interestingly, the MMR-proficient tumors failed to show a significant improvement with checkpoint inhibitors and have stimulated the search for potential strategies to improve the outcomes. In this context, SBRT, along with checkpoint inhibitors, has been evaluated for advanced colorectal cancers. The SABR-PDL1 (NCT02992912) study evaluated the combination of SBRT along with Atezolizumab in previously treated metastatic colorectal cancer patients. The OS and PFS were low, but an assessment of the immune context of these patients showed redirection of immune cells to the tumors as well as alteration of various immune-related biomarkers [127,128,129]. ILOC (NCT03101475) evaluated the overall immune response rate of lesions not treated by ablation, in patients with metastatic colorectal cancers who received immunotherapy and partial tumor ablation. Interestingly, no abscopal effect was seen in this study population [130].

Esophageal and gastric cancers are regularly treated with chemotherapy and radiation as well as surgery in the curative setting. Unfortunately, both cancer types are associated with a high risk of distant metastases and poor survival outcomes. PD-L1 expression is elevated in approximately 38% to 45% of esophageal and gastric cancers, and, prior to the immunotherapy era, was considered to be a poor prognostic factor. Subsequent studies have shown significant benefit from checkpoint inhibitors in the treatment of advanced cancers. The KEYNOTE-181 study reported prolonged OS with the use of Pembrolizumab when compared to second-line chemotherapy in advanced esophageal cancers [131]. This has encouraged researchers to explore novel ways to improve the therapeutic outcomes. The ESO-Shanghai 13 trial (NCT03904927) study evaluated the benefit of combining systemic treatment with SBRT for oligo-metastatic esophageal cancer. Patients received either systemic treatment and SBRT or systemic treatment alone. A total of 40% of the patients received immunotherapy with systemic treatments. At the time of analysis, there was a significant improvement in progression-free survival in the arm with local treatment (15.3 months vs. 6.4 months with systemic treatment alone) [72], providing some of the earliest evidence for the benefit of local treatment along with systemic treatment for this tumor subtype. Ongoing trials like the NCT07330583, ABIMMUNE (NCT03212469), NCT05183958, NCT05732662, ESO-Shangahai19 (NCT05760391), Shanghai20 (NCT06190782) and NCT05626569 trials are evaluating the benefit of the combination of checkpoint inhibitors with SBRT.

Gastrointestinal malignancies thus constitute a mixed set of cancers with regard to response to a combination of SBRT and immunotherapy. They are also some of the most common types of cancers and quite often the most aggressive and, hence, there will be considerable interest in evaluating strategies to improve outcomes. Treatments targeting the canonical immune pathways as well as emerging pathways are likely to shed more light on the utility of combining SBRT and immunotherapy in this group of patients.

## 12. Breast Cancer

Checkpoint inhibitors have been noted to have promising activity in breast cancers, especially in the triple-negative setting (TNBC). The Impassion130 (NCT02425891) and KEYNOTE-355 (NCT02819518) studies [132,133] reported a benefit for checkpoint inhibitors in the metastatic setting of TNBC, and the I-SPY2(NCT01042379) and KEYNOTE-522 (NCT03036488) reported promising outcomes in the setting of early-stage TNBC [134,135]. While acknowledging that the number of studies evaluating the benefit of the combination of radiation with checkpoint inhibitors is modest, the few studies reported in the metastatic setting failed to show a clear benefit with the combination [73,74,75].

Several ongoing trials are exploring this approach in both the metastatic and non-metastatic settings of breast cancer. The TROG 17.05 AZTEC (NCT03464942) is recruiting metastatic TNBC patients with brain metastases for treatment with the combination of Atezolizumab and SBRT. A number of studies are also evaluating the addition of further targeted agents with SBRT and checkpoint inhibitors to augment the synergy. The AGADIR study (NCT03915678) combines Pembrolizumab with SBRT and the addition of a Toll-like receptor agonist (BDB001) for the treatment of metastases. NCT04683679 and TARA (NCT04690855) are two studies currently recruiting metastatic TNBC patients for treatment with SBRT and Pembrolizumab and Atezolizumab, respectively, along with the addition of a PARP inhibitor. Interestingly, there has also been a renewed interest in the use of the combination treatment approach in the neoadjuvant treatment setting of TNBC, with the NCT06165900 and the NCT06401005 being two recently opened studies that are evaluating the use of Adebrelimab and Cadonilimab respectively, along with chemotherapy and SBRT. Thus, a number of trials evaluating the combination of SBRT and immunotherapy are planned or ongoing, and the findings reported will decide the future of this treatment strategy. Considering the established management approach for this cancer, the likely benefit from combination strategies will be most relevant in the metastatic setting, although the neoadjuvant trial data will be interesting to critically assess.

## 13. Toxicity of Combined Treatment

The toxicity associated with the combination of SBRT and immunotherapy has been studied in detail in multiple prospective studies that have been done in the last few years. Depending on the site of treatment, the tumor type and the dose of radiation used, the side effects differ, although the overall understanding is that of acceptable toxicity and absence of relative differences in toxicity when comparing SBRT alone with SBRT and immunotherapy. Grade 2 or higher toxicity is reported with SBRT alone when compared to other treatments, and immunotherapy is associated with its own potential complications, which are usually immune related. Hence, the potential toxicity of radiotherapy combined with immunotherapy cannot be discounted and requires further evaluation. The previously discussed study by Luke et al. evaluated the toxicity associated with the use of Pembrolizumab and SBRT in various cancer subtypes and cancer sites [51]. Dose limiting grade 3 or higher immunotherapy related toxicity was noted in less than 12% of the treated group, and none of the patients required a reduction in radiation dose. The PEMBRO-RT study showed no difference in toxicity between the Pembrolizumab arm and the combined SBRT + Pembrolizumab arm [56]. Similarly, no additional toxicity was noted in the Bauml et al. study either [55]. The NRG-LU004 study is currently ongoing and will examine the toxicity profile associated with the use of immunotherapy in lung cancers. A review of the studies that evaluated the combination treatment approach for brain metastases showed that the proportion of grade 3–4 neurological adverse events ranged between 0-13%. A pooled analysis of multiple studies that evaluated treatment of brain metastases from NSCLC and melanomas was recently reported and noted that the risk of radiation necrosis or treatment related toxicity was higher in the combined treatment group [85]. Interestingly, a subgroup analysis showed that this toxicity was noted in the melanoma group and not in the NSCLC patients. Another retrospective review of patients who received SRS/SBRT and checkpoint inhibitors reported evidence of radionecrosis in 27% of the patients evaluated, although this did not have an impact on the OS of these patients [136]. In the pelvic treatment setting, a phase I trial of Pembrolizumab and hypofractionated radiation for muscle invasive bladder cancer reported a significant risk for dose limiting toxicity [137], while on the other hand the combination of Ipilimumab and radiation for pre-treated metastatic castrate-resistant prostate cancer did not show an increase in pelvic toxicity. Hence, although the combination treatment approach does appear well tolerated in many of the patients, more clinical data is required to assess the risks associated with this treatment. It is likely that the cancer type and the site being treated will play an important role in defining the toxicity associated with the combination of immunotherapy and hypofractionated radiation.

## 14. Looking Forward

With the growing interest in the combination of immunotherapy with radiotherapy, there are a number of ongoing as well as proposed clinical trials examining the efficacy of this strategy in multiple cancer sub-types and stages. It will be worthwhile noting that tumors with higher mutational burden are more likely to respond to the combined treatment protocols [138]. The optimal radiation modality and the treatment dose and fractionation may also be variable depending on the tumor type. In this context, stereotactic hypofractionated radiation along with immunotherapy appears to be a promising treatment strategy with a reasonable safety profile. A number of factors have been identified that can impact outcomes with the combination of SBRT and immunotherapy (Table 3). Questions remain regarding the volume of disease requiring local treatment, the sequencing of each treatment modality and the number of sites that require treatment to achieve optimal outcomes. Potential strategies to improve the benefit of treatment include identifying the optimal patient population based on tumor characteristics, use of novel tumor microenvironment modulating agents, metastasis directed SBRT, and advanced image-guided treatment. As we see more mature data from the ongoing studies, we are likely to have better answers for these queries and potentially have a positive impact on patient treatment outcomes with this combination therapy.

## 15. Conclusions

Immunotherapy has helped make significant inroads into the management of various cancer subtypes, and there is growing awareness that the immune system plays an extremely important role in the fight against cancer. But there are limitations to the effectiveness of this strategy, and combining immunotherapy with SBRT has demonstrated promise. That said, the clinical benefit is highly context-dependent and, for many malignancies, this strategy remains investigational. This approach is likely of greater relevance to cancers where SBRT can improve the inherent immunogenic potential and should be explored in future trials.

## Figures and Tables

**Table 1:** Prospective trials evaluating the combination of Immunotherapy and SBRT.

Cancer Type	Study Title/ID	Study Details	*n*	RT Dose	Outcomes			Toxicity-≥gr3
					mOS	mPFS/EFS	ORR	
Lung	NCT02316002 [55]	Phase II Study of Pembrolizumab After Curative Intent Treatment for Oligometastatic Non-Small Cell Lung Cancer	45 (30 had SBRT)		41.6 mo (27–56.2 mo)	19.1 mo (9.4–28.7 mo)		
	PEMBRO-RT (NCT02492568) [56]	Randomized Phase II, 2-arm Study of Pembrolizumab After High-Dose Radiation (SBRT) Versus Pembrolizumab Alone in Patients With Advanced Non-small Cell Lung Cancer	76	24 Gy in 3 fractions	15.9 mo vs. 7.6 mo (HR 0.66; CI = 0.37–1.18; *p* = 0.16)	6.6 mo vs. 1.9 mo (HR 0.71, CI = 0.42–1.18; *p* = 0.19)	36% vs. 18% (*p* = 0.07)	
	NCT02239900 [57,58]	Phase I/II Trial of Ipilimumab (Immunotherapy) and Hypofractionated Stereotactic Radiation Therapy in Patients With Advanced Solid Malignancies	35 and 106	50 Gy in 4 fractions or 60 Gy in 10 fractions concurrently or sequentially with systemic treatment		2.9 mo		36%
	KEYNOTE-867 (NCT03924869) [59]	Phase 3, Randomized, Placebo-Controlled Clinical Study to Evaluate the Safety and Efficacy of Stereotactic Body Radiotherapy (SBRT) With or Without Pembrolizumab (MK-3475) in Participants With Unresected Stages I or II Non-Small Cell Lung Cancer (NSCLC) (KEYNOTE-867)	448	45–70 Gy in 3–8 fractions		EFS of 31.2 mo vs. 28.3 mo (HR 0.92, CI = 0.69–1.24, *p* = 0.29)		20.4%
	I-SABR (NCT03110978) [60]	Phase II Randomized Clinical Trials Comparing Immunotherapy Plus Stereotactic Ablative Radiotherapy (I-SABR) Versus SABR Alone for Stage I, Selected Stage IIa, or Isolated Lung Parenchymal Recurrent Non-Small Cell Lung Cancer: I-SABR	141	50–70 Gy in 4 to 10 fractions		77 vs. 56% EFS at 4 years (HR 0.38, CI = 0.19–0.75; *p* = 0.0056)		15%
	ASTEROID (NCT03446547) [61]	Randomized Phase II Trial With Durvalumab Following SBRT in Patients With Stage I Non-Small Cell Lung Cancer (NSCLC)	104	No survival difference at 33 months		Numerically fewer progression events (*p* = 0.054)		
Melanoma	NCT02821182 [62]	Phase II Trial of Stereotactic Body Radiotherapy With Concurrent Anti-PD1 Treatment in Metastatic Melanoma.	20	24 Gy in 3 fractions			45%	
	NCT01557114 [63]	Phase I trial to determine the side effects and best dose of radiation therapy administered in combination with ipilimumab.	19	9, 15, 18 and 24 Gy	0.9 mo (0.5–2.0 mo)	0.4 mo (0.2–1.4 mo)	31%	MTD 9 Gy
Renal cell carcinoma	NIVES (NCT03469713) [64]	Nivolumab and SBRT in II and III Line of Patients With Metastatic Renal Cell Carcinoma (mRCC)	69	10 Gy in 3 fractions	20 mo	5.6 mo (2.9–7.1 mo)	ORR 17% and DCR 55%	
	RADVAX RCC (NCT03065179) [65]	Phase II Trial of Stereotactic Body Radiation Therapy in Combination With Nivolumab Plus Ipilimumab in Patients With Metastatic Renal Cell Cancer	29	50 Gy in 5 fractions			56%	
	RAPPORT (NCT02855203) [66]	Single arm phase I/II trial of Stereotactic Radiotherapy and Pembrolizumab for Oligometastatic Renal Tumors	30	20 Gy in 1 fraction or 30 Gy in 10 fractions	74% (53–87%) OS at 2 years	45% (27–62%) PFS at 2 years	FFLP at 2 yr was 92% (80–90%). ORR was 63% (44–80%) and DCR was 83% (65–94%).	13%
Prostate	CA184-043 (NCT00861614) [67,68]	A Randomized, Double-Blind, Phase 3 Trial Comparing Ipilimumab vs. Placebo Following Radiotherapy in Subjects With Castration Resistant Prostate Cancer That Have Received Prior Treatment With Docetaxel	799	8 Gy in 1 fraction	2 yr (25.2% vs. 16.6%), 3 yr (15.3% vs. 7.9%), 4 yr (10.1% vs. 3.3%), and 5 yr (7.9% vs. 2.7%)	4 vs. 3.1 mo (HR 0.70, CI = 0.61–0.82, *p* < 0.0001)		26% vs. 3%
Liver	PEMRAD (NCT03316872) [69]	Pembrolizumab and Stereotactic Radiotherapy Combined in Subjects With Advanced Hepatocellular Carcinoma-A Phase II Study	18	25–50 Gy in 5 fractions	12.6 mo (5.7–25.8 mo)	5.4 mo (2.8–9.9 mo)	41% (18–67%)	
	NCT03203304 [70]	Phase I Study of Stereotactic Body Radiotherapy (SBRT) Followed by Nivolumab or Ipilimumab With Nivolumab in Unresectable Hepatocellular Carcinoma	7 vs. 6	40 Gy in 5 fractions	41.6 (4.5 − x) vs. 4.7 mo (2.0–16.2 mo)	11.6(4.5 − x) vs. 2.7 mo (1.3–4.7 mo)	57% (23–87%) vs. 0% (0–39%)	
	START-FIT (NCT03817736) [71]	Sequential Trans Arterial Chemoembolization and SBRT Followed by Immunotherapy for downstaging hepatocellular carcinoma for Hepatectomy (START-FIT)	33	27.5–40 Gy in 5 fractions for the SBRT group			42% radiologic CR	33%
Esophagus	ESO-Shanghai 13 (NCT03904927) [72]	Randomized phase II trial evaluating role of Local Therapy for Patients with Oligorecurrent and Oligometastatic Esophageal Squamous Cell Carcinoma After Radical Treatment	104	30–50 Gy in 3–10 fractions depending of site of treatment		15.3 mo vs. 6.4 mo (HR 0.26, CI 0.16–0.42, *p* < 0.0001)		43% vs. 41% (*p* = 0.538)
Breast	TONIC (NCT02499367) [73]	Adaptive Phase II Randomized Non-comparative Trial of Nivolumab After Induction Treatment in Triple-negative Breast Cancer (TNBC) Patients	70	24 Gy in 3 fractions		1.9 mo (1.8–2.0)	20% (11–31%) in Pd-1 blockage arm	3%
	NCT02730130 [74]	Single Arm Phase II Study to Assess the Efficacy of Pembrolizumab Plus Radiotherapy in Metastatic Triple Negative Breast Cancer Patients	17	30 Gy in 5 fractions			17.6% (4.7–44.2%)	17.6%
	NCT03366844 [75]	Preoperative Combination of Pembrolizumab and Radiation Therapy in Patients With Operable Breast Cancer	8	20 Gy in 5 fractions	2.9 mo (0.9–3.6%)	1.4 mo (0.4–2.1 mo)	0%	

PFS: Progression-Free Survival, OS: Overall Survival, ORR: Objective Response Rate, EFS: Event-Free Survival, DCR: Disease Control rate, FFLP: Freedom From local Progression, RT: Radiation therapy, SBRT: Stereotactic Body Radiation Treatment, IO: Immunotherapy.

**Table 2:** Ongoing studies evaluating the benefit of the combination of SBRT and immunotherapy.

Tumor Site	Study Title/ID	Study Details	Primary Endpoint	Status and Results
Lung	NRG-LU002 (NCT03137771)	Phase II/III trial evaluating maintenance chemotherapy with or without local consolidation therapy in stage IV NSCLC	Phase II: PFS Phase III: Overall survival	Active, not recruiting
	LONESTAR (NCT03391869)	Phase III trial with nivolumab and ipilimumab with or without local consolidation therapy in patients with stage IV NSCLC	Overall Survival	Active, not recruiting
	STICk-IM-NSCLC (NCT04650490)	Randomized, 2-arm, phase II study to determine the effect, of the timing of stereotactic radiosurgery (SRS) relative to immune checkpoint inhibitor (IO) therapy	Intracranial PFS	Withdrawn due to inadequate enrollment
	STRAITLUC (NCT04787185)	Observational trial evaluating patients with brain metastases from NSCLC who are candidates for radiosurgery or fractionated stereotactic radiation in course of immunotherapy	Evaluation of toxicity related to the combination of radiotherapy and immunotherapy	
	MIGRAINE (NCT04427228)	Randomized trIal of Single Versus Multifraction Radiosurgery on Immunotherapy	To determine if Multi-Fraction SRS will decrease the rate of radionecrosis when compared to single-fraction SRS for patients with small metastases and are on immunotherapy.	Withdrawn due to inadequate enrollment
	PACIFIC-4 (NCT03833154)	Efficacy and safety of durvalumab combined with SBRT in patients with stage I/II unresectable NSCLC	Progression-Free Survival	Active and ongoing
	KEYNOTE-867 (NCT03924869)	Phase 3, randomized, placebo-controlled study of stereotactic body radiotherapy (SBRT) with or without pembrolizumab in patients with unresected stage I or II non–small cell lung cancer (NSCLC)	Event-Free Survival	Discontinued in 2024 after interim analysis showed lack of a statistically significant event-free survival advantage on the experimental arm
	I-SABR (NCT03110978)	Randomized phase 2 trial of SABR alone compared with SABR with immunotherapy (I-SABR) for people with early-stage NSCLC.	Event-Free Survival	Compared with SABR alone, I-SABR significantly improved event-free survival at 4 years in people with early-stage treatment-naive or lung parenchymal recurrent node-negative NSCLC
	ASTEROID (NCT03446547)	Randomized multicenter open-label phase 2 study comparing SBRT alone versus SBRT followed by adjuvant treatment with durvalumab.	Time to progression (TTP).	Ongoing
	BR-38 (NCT06686771)	Randomized trial evaluating the consolidative Use of Radiotherapy to Block Oligoprogression In Patients With Metastatic Non-Small-Cell Lung Cancer	PFS and OS at 5.5 years	Ongoing
Melanoma	ABC-X (NCT03340129)	Phase II, open label, randomized trial of ipilimumab and nivolumab with concurrent intracranial stereotactic radiotherapy versus ipilimumab and nivolumab alone in patients with asymptomatic, untreated melanoma brain metastases	Neurologic specific survival (NSS) at 12 months	Ongoing
	HAMMER (NCT05169957)	Phase 1 trial of feasibility of liver stereotactic body radiation therapy (SBRT) given in combination with systemic therapy (ipilimumab and nivolumab) in adults with metastatic melanoma with liver metastases who are at significant risk of not benefiting from systemic therapy alone	Percentage of patients who receive all planned radiotherapy.	Completed-results awaited
Renal Cell Carcinoma	NCT02781506	Phase 2 trial with SBRT to multiple metastatic sites concurrently administered with Nivolumab for patients with metastatic clear cell renal cell cancer who have failed at least one anti-angiogenic therapy.	RR (response rate) of treatment with Nivolumab by the concurrent administration of SBRT	Terminated-due to change in practice landscape. Data from treated patients awaited.
	NCT02855203	Phase 1/2 trial evaluating safety, efficacy and biological effects of combining pembrolizumab (MK-3475) an antibody targeted against anti-programmed cell death 1 (PD-1), with stereotactic ablative body radiotherapy (SABR) for oligometastatic renal cell carcinoma (RCC)	Grade 3 Treatment Related Adverse Events as determined using CTCAE.	Completed
	NCT02599779	A phase II proof of concept, multi-center, safety and efficacy study to investigate if a treatment strategy where SBRT given with pembrolizumab is sufficiently active to warrant further investigation in randomized phase II or III studies	Progression-Free Survival	Completed-results awaited.
Prostate and bladder	POSTCARD (NCT03795207)	A Randomized Phase II Trial of Stereotactic Body Radiation Therapy (SBRT) With or Without Durvalumab (MEDI4736) in Oligometastatic Recurrent Hormone Sensitive Prostate Cancer Patients	Two-year progression-free survival	Active, but not recruiting
	ASTRA (NCT07413523)	Randomized trial of standard of care with or without metastases-directed SBRT in patients affected by oligometastatic urothelial carcinoma: ASTRA Trial	Local control	Active-not yet recruiting
Liver	NCT03482102	Phase II Trial of Durvalumab (MEDI4736) and Tremelimumab and Radiation Therapy in Hepatocellular Carcinoma and Biliary Tract Cancer	Best overall response rate	Active and recruiting
	NCT03203304	Phase I Study of Stereotactic Body Radiotherapy (SBRT) Followed by Nivolumab or Ipilimumab With Nivolumab in Unresectable Hepatocellular Carcinoma	Number of participants with adverse events	Study stopped due to poor accrual
	NCT03316872	Phase 2 study to assess the systemic efficacy of combined SBRT and pembrolizumab in subjects with advanced HCC who have experienced disease progression after previous therapy	Overall response rate	Study stopped due to change in practice. Study results show a high overall response rate (41%), especially in patients with macrovascular invasion (45%)
	NRG GI 012/HELIO-RT (NCT07166406)	Phase III trial comparing the effect of immunotherapy with stereotactic body radiation therapy (SBRT) to immunotherapy alone in treating patients with advanced hepatocellular cancer with macrovascular invasion	Overall survival	Active and recruiting
Pancreas	NCT03767582	Phase I/II Trial of Combination Immunotherapy With Nivolumab and a CCR2/CCR5 Dual Antagonist (BMS-813160) With or Without GVAX Following Chemotherapy and Radiotherapy for Locally Advanced Pancreatic Ductal Adenocarcinomas (PDACs)	1. Number of Participants experiencing study drug-related toxicities. 2. Percentage of participants treated with immunotherapy who achieve an immune response	Phase 1 study results show that the combination is safe and does not delay surgery. Phase 2 results awaited.
	NCT05088889	Single arm study, which aims to evaluate the efficacy and safety of combination therapy with SBRT, nivolumab and ipilimumab as a maintenance regimen following first line induction chemotherapy in patients with metastatic pancreatic cancer.	Objective tumor response rate	Pilot study completed. Disease control rates of 90%
	INFLUENCE (NCT05116917)	Nivolumab, Ipilimumab and Radiation in Combination With Influenza Vaccine in Patients With Pancreatic Cancer	Objective response rate	Study terminated based on the prespecified interim analysis that showed the predictive probability was below 10% for the prespecified endpoint.
	EMPIRE (NCT06843551)	Phase 2 Single Arm Trial of Stereotactic Body Radiation Therapy Followed by Dual Immune Checkpoint Inhibition for Patients With Metastatic Pancreatic Ductal Adenocarcinoma	Clinical benefit Rate	Ongoing
	PREOPANC-5 (NCT06384560)	Neoadjuvant Triple Treatment With mFOLFIRINOX, Pembrolizumab and SBRT in Patients With (Borderline) Resectable Pancreatic Cancer	Progression-Free Survival	Ongoing
Colorectal and other GI cancers	SABR-PDL1 (NCT02992912)	Phase II Study to Assess the Efficacy of the Anti-PD-L1 Antibody Atezolizumab Administered With SBRT in Patients With Metastatic Tumors	Progression-Free Survival	Active, not recruiting. No PFS benefit seen, but improved immune response noted
	ILOC (NCT03101475)	Phase II of Immunotherapy Plus Local Tumor Ablation (RFA or Stereotactic Radiotherapy) in Patients With Colorectal Cancer Liver Metastases	Best overall immune response rate (iBOR) of lesions not treated by ablation/radiotherapy including the extrahepatic lesions according to iRECIST	Completed-No response in untreated areas.
	NCT07330583	Phase II Single Arm Study to Evaluate Stereotactic Body Radiation Therapy (SBRT) and Immunotherapy for Management of Patients With Oligometastatic Esophageal Cancer	PFS	Not yet recruiting
	ABIMMUNE (NCT03212469)	Phase I/II Study Evaluating the Safety and Clinical Activity of Anti-PDL1 (Durvalumab [MEDI4736]) + Anti CTLA-4 (Tremelimumab) Antibodies Administrated in Combination With Stereotactic Body Radiotherapy (SBRT) in Patients With Metastatic Squamous Cell Carcinoma of Head and Neck, Lung, Oesophageus, Cervix, Vagina, Vulva or Anus	Dose limiting toxicity	Completed-data awaited
	NCT05732662	SBRT Combined With PD-1/CTLA-4 Dual Antibody to Overcome Anti-PD-1 Resistant in Relapsed or Metastatic Esophageal Squamous Cell Carcinoma	Objective response rate	Not yet recruiting
	NCT05760391 (ESO-Shanghai19)	Single arm study evaluating the combination and timing of Immunotherapy With Radiotherapy in Patients With Advanced Esophageal Squamous Cell Carcinoma.	OS from start of 1st line treatment in metastatic ESCC	Recruiting
	NCT06190782 (ESO-Shanghai20)	Phase III Randomized-controlled Study of PD-1 Inhibitor Combined With Local Therapy in Patients With Oligometastatic Esophageal Squamous Cell Carcinoma	PFS difference in PD-1 inhibitor + radiotherapy and PD-1 inhibitor alone	Recruiting
	NCT05626569	Phase 2 Study of Anti-PD-1 Immunotherapy Combined With Stereotactic Body Radiation Therapy for Patients With Oligometastatic Esophageal Squamous Cell Carcinoma	PFS at 1 year	Ongoing
Breast	TROG 17.05 AZTEC (NCT03464942)	Randomized Phase II Trial Comparing the Efficacy of Single-fraction (20 Gy in 1 fraction) or Multi-fraction SBRT (24 Gy in 3 fractions) With Atezolizumab in patients with advanced triple negative breast cancer	Progression-Free Survival	Study completed. The median PFS for the 20 Gy arm was 2.5 (90% CI: 1.7–4.5) months, 3.1 (90% CI: 1.8–3.9) months for the 24 Gy arm. OS data awaited.
	AGADIR (NCT03915678)	Basket trial concept to independently and simultaneously assess the effects of the association of atezolizumab + BDB001 + radiotherapy in multiple solid tumors.	Antitumor activity assessed in terms of disease control rate within 24 weeks of treatment onset and defined as the proportion of patients with complete response (CR), partial response (PR) or stable disease (SD) observed within 24 weeks of treatment onset (while treated with the investigational product), based on RECIST 1.1 criteria.	Recruiting
	NCT04683679	Phase II Study of Pembrolizumab and Ablative Radiotherapy With or Without Olaparib in Metastatic Triple-Negative or Hormone-Receptor Positive/Her2 Negative Breast Cancers: Initial Test Cohorts of a Platform Trial to Sequentially Investigate Immunotherapy Combinations for the Augmentation of Immune Responses	Overall response rate	Recruiting
	NCT06238921	Phase I/II Study of Stereotactic Radiation and Sacituzumab Govitecan With Zimberelimab in the Management of Metastatic Triple Negative Breast Cancer With Brain Metastases (TARGET-TNBC)	Phase 1: Neurotoxicity Phase 2: PFS	Recruiting
	NCT06165900	A Multicenter, Randomized Trial of Stereotactic Radiotherapy Combined With Adebrelimab and TCb (Nab-paclitaxel + Carboplatin) Versus Adebrelimab Combined With TCb (Nab-paclitaxel + Carboplatin) in Neoadjuvant Treatment of Triple-negative Breast Cancer	Pathologic complete response	Recruiting
	NCT02499367	Adaptive Phase II Randomized Non-comparative Trial of Nivolumab After Induction Treatment in Triple-negative Breast Cancer (TNBC) Patients: TONIC-trial. Radiation therapy and immunotherapy to be compared against chemotherapy and immunotherapy in metastatic TNBC	PFS	Stage 1 component of trial reported feasibility of approach.
	NCT06401005	A Single-arm, Open, Phase II Clinical Study of SBRT, Chemotherapy, and Cadonilimab (AK104) Neoadjuvant Therapy for Triple-negative Breast Cancer (TNBC)	Pathologic complete response	Recruiting

PFS: Progression-Free Survival, OS: Overall Survival, ORR: Objective Response Rate, EFS: Event-Free Survival, RT: Radiation therapy, SBRT: Stereotactic Body Radiation Treatment, IO: Immunotherapy.

**Table 3:** Determinants of efficacy of the combination of SBRT and immunotherapy.

Determinant	Parameter	Detail
Radiation dose and fractionation	Ablative dose	Ablative doses required to trigger cell death and release of tumor associated antigens
	Pathway activation	Cross presentation of antigens and activation of cytotoxic T-cells.
	Immune sparing	Minimizing damage and preservation of immune cells
Timing and sequencing of treatments	Timing of immunotherapy before, during or after SBRT	Studies suggest that starting immunotherapy after SBRT yields the best systemic outcomes.
	Immune system priming	Optimal sequencing leads to radiation-induced antigen release and immune priming
Microenvironment and host immunity	Immunological state of tumor	“Hot” tumors with existing T cell infiltration show good response. “Cold” tumors show poor response to immunotherapy, which can be abrogated by SBRT
	Host immune system	Robust and active immune system is essential for achieving a good outcome
	Immunosuppressive cells	Presence of immunosuppressive cells like T-regs and tumor associated macrophages can blunt effect of immunotherapy. High-dose SBRT can potentially deplete the immunosuppressive cells and re-engage the immunotherapy related response.
Abscopal effect	Tumor type	Highly dependent on tumor type and variable
	Tumor burden and heterogeneity	Abscopal response is often limited by a high degree of intra-tumoral heterogeneity and massive overall tumor burden

## Data Availability

No new data were created or analyzed in this study. Data sharing is not applicable to this article.
